# Activated Carbon and Diatomite as Filtration Materials for Nutrient Removal from Stormwater

**DOI:** 10.3390/ma18204742

**Published:** 2025-10-16

**Authors:** Agnieszka Grela, Justyna Pamuła, Karolina Łach, Izabela Godyń, Dagmara Malina, Damian Grela

**Affiliations:** 1 Faculty of Environmental Engineering and Energy, Cracow University of Technology, Warszawska 24, 31-155 Cracow, Poland; agnieszka.grela@pk.edu.pl (A.G.); karolina.lach@pk.edu.pl (K.Ł.); izabela.godyn@pk.edu.pl (I.G.); 2 Faculty of Chemical Engineering and Technology, Cracow University of Technology, Warszawska 24, 31-155 Cracow, Poland; dagmara.malina@pk.edu.pl; 3 Faculty of Electrical and Computer Engineering, Cracow University of Technology, Warszawska 24, 31-155 Cracow, Poland; damian.grela@pk.edu.pl

**Keywords:** granular activated carbon (GAC), diatomite, stormwater treatment, rain garden, bioretention, phosphates, nitrates

## Abstract

Activated carbon used as one of the layers of a rain garden may be a promising solution for removing nutrients (nitrogen and phosphorus compounds) from stormwater runoff. Progressive urbanization degrades the quality of stormwater that reaches water collectors. Rain gardens are a potential solution—nature-based systems that retain, infiltrate, and purify stormwater. The aim of this study was to evaluate the effectiveness of a model rain garden in the form of retention columns, depending on the composition of the filling material and the conditions of the simulation. The base column was filled with sand, gravel, and dolomite. The next two columns were enriched with diatomite, in a weight ratio to sand of 1:4 and 1:2, respectively. The experiment was based on four scenarios: (1) 30 min of heavy rain, (2) 2 h of rain after a drought, (3) during standard operation, and (4) with modification of the filtration material. This modification consisted of a uniform addition of granular activated carbon (GAC), which was intended to influence the column performance. The characteristics of the activated carbon were determined using XRD, SEM-EDS, and BET analysis. Pollutant concentrations were determined using a spectrophotometer and ion-selective electrodes. The analyses confirm the significant impact of the column filling materials on the efficiency of nutrient removal from stormwater, achieving even complete removal of phosphate ions, while nitrate ions were removed at a level of almost 40% and ammonium ions at >90%.

## 1. Introduction

The European Commission has introduced the concept of Nature-Based Solutions (NBSs), defined as solutions inspired and supported by nature that deliver environmental, social, and economic benefits and increase the resilience of cities to extreme weather events [1]. NBSs include, among others, rain gardens, which serve as elements of urban stormwater management systems [2]. They are an exemplary solution supporting the adaptation of urban areas to heavy rainfall and have a positive impact on the social environment [3].

A rain garden is a shallow depression or special container filled with a permeable substrate, designed to control surface runoff and improve water retention [4]. Such systems reduce the volume and peak flow of stormwater, reducing the risk of flooding and sewer overload [5].

Rain gardens not only retain water but also effectively purify it of suspended solids, biogenic compounds (N and P), heavy metals, polycyclic aromatic hydrocarbons, fats, and microplastics larger than 10 µm [6,7,8,9,10,11,12,13]. Excess nitrogen and phosphorus in urban surface waters lead to eutrophication and cyanobacterial blooms, causing ecosystem degradation [14,15,16]. In rain gardens, nitrogen purification is based on nitrification and denitrification, while phosphorus is removed through sorption in beds rich in Ca, Al, and Fe or through filtration of solid particles [17,18,19,20,21,22,23,24,25].

The efficiency of purification depends on the type of filtration material. In addition to sand, sorptive additives, such as diatomite, zeolite, biochar, and activated carbon, are used, which increase the effectiveness of removing nitrogen, phosphorus, and other pollutants [7,9,10,25,26,27,28,29,30,31,32,33,34,35,36,37,38,39].

Proper design of a rain garden is crucial for the effectiveness of this system. Wang et al. [40] emphasized that optimizing designs for climates and ensuring long-term performance remain a challenge. As reported by Liu et al. [25], the effectiveness of rain gardens is primarily determined by media types and pollutant species, rather than pollutant influent concentration, operating time, and inflow hydraulic loading. Therefore, the material that fills rain gardens is of great importance. Table 1 presents a literature review regarding rain garden design, considering three key criteria: water retention capacity, pollutant removal capability, and safety for the aquatic environment.

Water retention capacity is the potential of a rain garden to retain, store, and infiltrate stormwater. It is an important feature that influences the effectiveness of rain gardens as stormwater management tools in urban environments [43]. The retention capacity of a rain garden is defined as the ability to retain stormwater from sealed surfaces through infiltration, evaporation, and short-term retention, which results in a reduction in the volume of surface runoff [44]. Studies conducted in the USA indicate that a well-designed rain garden with an area of 30 m^2^ can retain up to 75–90% of the annual rainfall volume from a roof of approximately 100 m^2^ [45]. Similar reductions in runoff volume have been observed in rain garden studies: built with river sand 88–98% [6], with soil and mulch around 80% [26], with the addition of GAC 63–97% and biochar 65–100% (depending on the return period of rainfall) [27], modification of the filler (with zeolite, steel slag, biochar) can increased the runoff and peak flow reduction rates by 12.2–29.4% and 31.5–63.4% [46]. Peak flow reduction was also studied depending on the type of garden construction, demonstrating a 56% and 80% reduction in monophasic and biphasic rain gardens, respectively [28].

The ability of rain gardens to retain pollutants refers to their capacity to reduce the load of harmful substances found in stormwater runoff from paved surfaces. This process is primarily based on physical filtering, sorption, biodegradation, and bioaccumulation within the filtration layers and the root zone of plants [29]. Rain gardens effectively remove pollutants, such as total suspended solids, nitrogen, phosphorus, and heavy metals, from surface runoff through a combination of sedimentation, filtration, plant uptake, and microbiological processes [30]. Rain gardens that use only sand as a filtering material have limited effectiveness in removing pollutants from stormwater [7,25]. Numerous studies show that the use of sorptive additives, such as diatomite [7], zeolite [25,31,32], carbon in the form of mulch, biochar, or wood chips [9,26,32,33,34,35,36,37], or GAC [10,27,31,33,38,39], significantly increases the efficiency of the treatment processes, including the removal of biogenic compounds.

Water environment safety in the context of rain gardens means that their construction and operation ensure that the pollutants collected in them are not secondarily washed out into the aquatic environment (e.g., rivers, canals, groundwater) during subsequent rainfall or system saturation [47]. The environmental safety of rain gardens refers to their ability to permanently retain pollutants without the risk of their remobilization (e.g., during intense rainfall), which protects water recipients from secondary pollution [48]. Jeon et al. [6] conducted a 5-year study and showed that filtration and bioremediation mechanisms minimize the leaching of pollutants, but they emphasized that the devices should be properly maintained. Studies also indicate leaching of pollutants, e.g., Chen et al. [26] showed negative reductions in EMC, indicating periodic leaching of components (mainly nitrogen and COD). Gilchrist et al. [33] studied nitrogen removal in rain gardens and demonstrated that leaching processes can be initiated by high nitrogen loads. An increase in NO_3_-N concentration was also observed by Davis et al. [8]; in the case of phosphorus, they drew attention to desorption and release of P in the situation of pH change, and they also pointed out that due to the accumulation of phosphorus, its removal should be carried out by cutting and harvesting vegetation that absorbs nutrients from the substrate. Huang et al. [32] selected the optimal composition of filtering materials and proposed a mixture that created a self-regulating remediation system, which not only increased the effectiveness of removing many pollutants but also reduced the risk of secondary pollution.

Existing research has focused mainly on retention and purification functions, as well as the selection of filtration layers and sorbent additives. However, the interactions between the filtration material and the effectiveness of nitrogen and phosphorus removal under variable rainfall and drought conditions remain insufficiently understood (Table 1).

This study attempts to modify the construction of a rain garden by adding locally available materials to the filtration bed: diatomite and activated carbon (GAC). This research covered various rainfall intensities and drought periods, allowing an assessment of the impact of additives on water retention and nutrient removal efficiency. The results may provide a basis for developing design guidelines that enable the creation of rain gardens with high efficiency, durability, and the ability to limit surface water eutrophication.

The aim of the research conducted by the authors was to check whether the addition of activated carbon, which is used in rain gardens, improves the effectiveness of nutrient removal. Additionally, continuing their research using diatomite [7], the authors assessed the impact of drought periods and changes in rainfall intensity on the performance of a rain garden with diatomite added in various proportions.

Table 1 presents an overview of the materials used in rain gardens, which were evaluated according to three criteria: water retention capacity, pollutant removal capability, and safety for the aquatic environment. The greatest consistency concerns criterion 2—pollutant removal—all materials presented in Table 1 [6,7,8,9,10,13,17,25,26,27,28,31,32,33,34,35,36,37,38,39,41,42] proved to be effective in removing pollutants.

Significantly larger differences were observed in the retention capacity—criterion 1. Systems with a well-balanced proportion of soil, sand, and organic additives (e.g., [6,17,26,32]) were characterized by a positive assessment in all criteria. In contrast, systems based mainly on sand or mineral materials, such as diatomite, dolomite, or ash, often did not meet the retention requirements (e.g., [7,9,28,39]). This suggests that the presence of an appropriate fine-grained fraction and organic components increases the water storage capacity.

A similar differentiation occurs in terms of environmental safety—criterion 3. Some materials, especially those with the addition of industrial waste or sewage sludge (e.g., [7,9,28,39]), did not meet this criterion. This may be due to the potential risk of the secondary release of pollutants. In turn, mixtures enriched with biochar, peat, or organic materials (e.g., [15,17,33]) received positive ratings, which confirms their stability and beneficial impact on the aquatic environment.

The approach presented in Table 1, using three criteria that were assessed, can be evaluated by indicating the advantages and disadvantages. The advantage of using mixed materials (soil, sand, organic additives, and biochar) is a good balance between retention capacity, pollutant removal, and environmental stability [6,15,17,26,32]. The advantages of using mineral materials (sands, gravels, dolomites, diatomites) include high filtration efficiency and simple construction, but there is limited water retention and the risk of leaching of mineral components [7,28]. The disadvantage of using industrial waste (e.g., SBAC—sludge-based activated carbon) is that, despite good sorption efficiency, it has low retention and environmental safety issues [39]. The advantages of additives such as biochar or zeolites include increased sorption capacity and often improved chemical stability of the system, but they require further research on long-term durability and regeneration [13,31,35,36].

After analyzing Table 1, the authors state that the best results are achieved by using multi-component mixtures that combine mineral materials (for filtration and structural stability) with organic and sorptive components (for retention and pollutant reduction). Configurations using biochar, peat, or other sorbents in combination with soil and sand have proven to be particularly effective [15,17,33]. Single-component materials or those based solely on industrial waste are characterized by limited effectiveness and may generate environmental risks [7,28,39]. Ultimately, to meet all three criteria, it is crucial to properly balance the filtration layers and select materials both in terms of hydrological properties and ecological safety.

## 2. Materials and Methods

### 2.1. Methodology for Testing GAC Properties

Activated carbon was characterized in terms of its physicochemical properties using the following instrumental techniques. X-ray diffraction (XRD) analyses were performed on a SmartLab SE (Rigaku, Tokyo, Japan) apparatus equipped with a horizontal HyPix-400 semiconductor (2D) X-ray detector and a Cu LFF anode tube. The XRD pattern was collected in the 2θ angle range between 5 and 80°, with a step width of 0.02°. The crystalline phases were identified by using the ICDD PDF-5 database [49].

Low-temperature nitrogen adsorption–desorption isotherms were measured at −196 °C using a multi-function adsorption instrument, surface area and porosity analyzer, Micromeritics ASAP 2020 (Micromeritics, Bruxelles, Belgium). Before the measurements, the activated carbon sample was degassed under vacuum at 250 °C for 12 h to ensure complete removal of impurities from the surface of the sample. Specific surface areas (*S_BET_*) were calculated using the Brunauer–Emmett–Teller method within the relative pressure of *p*/*p*° = 0.06–0.2. The total pore volume (*V_total_*) was determined using the single-point method at a relative pressure of *p*/*p*° = 0.978. The average pore diameter (*D*) was calculated by applying the BET formalism according to the formula *D_A_* = 4*V_total_*/*S_BET_*. Pore size distributions were calculated from the desorption branches of the isotherms using the Barrett–Joyner–Halenda (BJH) model.

The morphology of the activated carbon was analyzed using an Apreo 2 S LoVac scanning electron microscope (Thermo Fisher Scientific, Waltham, MA, USA) equipped with an EDS UltraDry and Octane Elect detectors (EDAX Ametek GmbH, Weiterstadt, Germany). The samples were sputter-coated with a 2.5 nm layer of gold in an argon atmosphere. The analysis was carried out under high-vacuum conditions.

### 2.2. Methodology for Determining Nutrients

The spectrophotometric method with ammonium molybdate according to Polish Standard PN-EN ISO 6878:2006 [50] was used for the determination of phosphorus compounds in all types of water, including seawater and runoff. Determination of phosphate ions by the phosphomolybdenum blue method is based on the formation of a complex compound in an acidic medium as a result of the reaction of orthophosphates with ammonium molybdate in the presence of antimony ions, from which phosphomolybdenum blue is formed after reduction with ascorbic acid. The intensity of the color induced is proportional to the orthophosphate content and is measured by spectrophotometric measurement of absorbance.

In this work, the phosphate content in the analyzed stormwater samples was confirmed by measuring the absorbance at 700 nm using a UV-Vis spectrophotometer (Evolution 220, ThermoScientific, Waltham, MA, USA) [51].

NO_3_^−^ and NH_4_^+^ ion concentrations were analyzed using ion-selective electrodes along with reference electrodes purchased from the company Detector (Raszyn, Polska). Detailed electrode characteristics are presented in Table 2.

Ion-selective electrodes are compatible with the CPI-601 Ionometer from Elmetron (Zabrze, Polska). The ionometer measures ion concentrations from 0 to 1000 g L^−1^, with a resolution of 0.01/0.1, and features automatic compensation for temperatures ranging from −5 to 110 °C [53].

Conductivity was analyzed using the CX-505 multifunctional meter from Elmetron (Zabrze, Polska). The operating range is 0 to 1999.9 mS cm^−1^. The meter automatically compensates for temperatures ranging from −5 to 70 °C [54].

### 2.3. Methodology of Conducting Experiments

Three filter columns, namely, C1, C2, and C3, were used for the experiments; a detailed description can be found in [7]. Each column was made of epoxy-coated plexiglass pipes and had an internal diameter of 140 mm. The columns contained, from bottom to top, a 30 cm layer of dolomite with a grain size declared by the manufacturer as 8–16 mm, followed by a 45 cm layer of pure sand (C1), sand with diatomite (C2 and C3) (sand granulation: 0.5–1.4 mm, diatomite granulation: 0.5–2 mm), and a 5 cm layer of gravel (2–8 mm) at the top. At a height of 20 cm from the bottom, an outlet was placed to allow water drainage from the columns. Columns with such filling were used in 3 experiments:

1. Experiment 1 (exp.1): three rainfall simulations lasting 30 min and with a height of about 32 mm (at 1–2-day intervals)—comparison of the effectiveness of diatomite addition [7].
2. Experiment 2 (exp.2): single rainfall simulation (120 min, 43 mm)—testing the effectiveness of the columns after a 3.5-month drought.
3. Experiment 3 (exp.3): three rainfall simulations (120 min, 43 mm) (with an interval of 1–2 days)—testing the effectiveness of the columns under less intense rain than in experiment 1.

**Figure 1:**
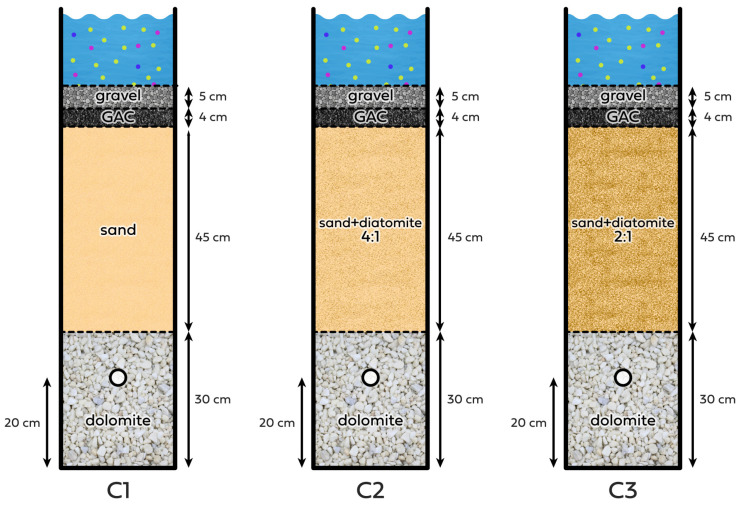
Schematic diagram of the C1, C2, and C3 structures. The circle at the bottom of each column symbolizes the outlet.

After conducting the above three experiments, the structure of the columns was modified. The gravel layer was removed from each column, 4 cm (340 g) of granular activated carbon (GAC) was added, and the gravel was poured back in (Figure 1). Granular activated carbon (GAC) of coconut origin with a granulation of 0.6–2.36 mm, purchased from Browin (Łódź, Poland), was used in this study. This is a typical commercial material, widely available in retail (e.g., in gardening stores), and not a specialized laboratory reagent, which allows the results to be related to practical applications in the construction of rain gardens. Coconut carbon is one of the most commonly used raw materials in the production of GAC due to its high carbon content, high mechanical hardness, and developed microporosity [55]. Activated carbon from coconut shells is also characterized by high chemical purity, which translates to a low ash content compared to charcoal or hard coal. According to the manufacturer’s recommendations, it should be added in an amount of 5–10%, which was performed. All materials used for the construction of the columns came from the local market. Then, experiment 4 was conducted:

Experiment 4 (exp.4): three simulated rainfall events (120 min, 43 mm) (at 1–2 day intervals)—testing the efficiency of the columns when the filtration media was enriched with a 4 cm layer of GAC.

In summary, a total of four experiments were conducted in three columns:

1. Experiment 1 (exp.1)—intense rain for 30 min;
2. Experiment 2 (exp.2)—less intense rain for 120 min after a drought period;
3. Experiment 3 (exp.3)—less intense rain for 120 min;
4. Experiment 4 (exp.4)—less intense rain for 120 min with enrichment of GAC filtration media.

For exp.1, 3, and 4, three simulations were conducted, while for exp.2, one simulation was performed, with quantity and duration in accordance with the assumptions described above. For each column, leachates were collected, and a representative sample of 100 mL was taken after 10, 20, 30, and 40 min from the start of the experiment for exp.1, and after 30, 60, 90, 120, and 150 min for exp.2, 3, and 4. Each time, a sample of model rain was also taken to control its quality.

The selection of precipitation was made based on the recommendations of the local drainage network administrator, who recommends designing stormwater management devices for precipitation with a frequency of occurrence of c = 10 years. For the durations selected for the experiment (30 and 120 min), precipitation amounts were read from the precipitation model developed for Cracow, which were 31.6 and 42.9 mm, respectively.

It was assumed that the garden would be sized at 6% of the drainage area. The columns have a diameter of 15 cm and an area of 176.71 cm^2^, which allows for the drainage of a catchment area of 0.2945 m^2^. With the drainage area thus determined, the columns were supplied with the following total amount of synthetic rain:

- A height of 31.6 mm, 30 min of rainfall, rainfall intensity of 310 mL min^−1^, and a total inflow to the column of 9.3 L^3^;
- A height of 42.9 mm, 120 min of rainfall, a rainfall intensity of 105 mL min^−1^, and a total inflow to the column of 12.6 L^3^.

The columns were supplied with synthetic rain in the amount specified above, with the following nutrient concentrations: NH_4_^+^ 5.0 mg L^−1^, NO_3_^−^ 15.0 mg L^−1^, and PO_4_^3−^ 2.0 mg L^−1^ [7].

During experiment 1 (exp.1), a representative sample of leachate with a volume of 100 mL was taken 10, 20, 30, and 40 min after the start of measurement. In experiments 2, 3, and 4 (exp.2, exp.3, exp.4), samples were taken every 30 min, starting from the 30th minute and ending at the 150th minute. For all samples, selected qualitative parameters were determined using the above-described laboratory equipment: a UV-Vis spectrophotometer, ion-selective electrodes, and a multifunctional meter.

## 3. Results

### 3.1. Characteristics of GAC

**Figure 2:**
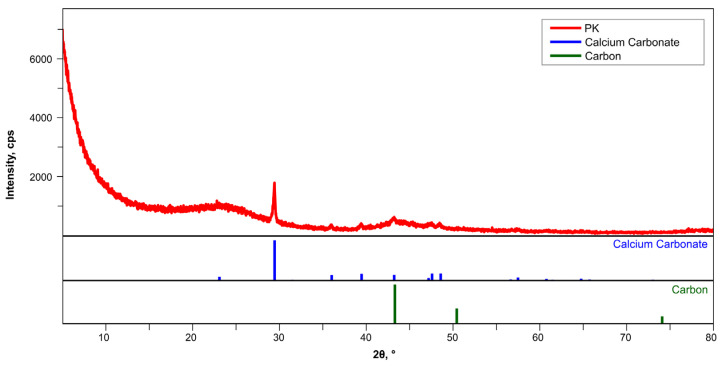
X-ray diffraction pattern for GAC.

The activated carbon used in this study is an amorphous or poorly crystalline material; therefore, it does not form sharp peaks in the XRD diffractogram (Figure 2). A broad background (so-called amorphous halo) can be observed, especially in the range of 15–30° 2θ, which is typical for activated carbon.

The presence of a peak at 29.5° 2θ indicates the presence of calcite (CaCO_3_), an inorganic additive, which was also confirmed by the match with the blue standard lines. In the sample, the amount of crystalline carbon is below the XRD detection limit or occurs as very finely divided, amorphous structures.

The porosimetry properties of the analyzed activated carbon derived from coconut shells are exhibited in Table 3. The surface area, total pore volume, and average pore diameter are 872.77 m^2^ g^−1^, 0.415 cm^3^ g^−1^, and 1.903 nm, respectively, which display expected adsorption properties when compared to other research [56,57,58,59,60,61]. According to IUPAC classification, pores with a diameter below 2 nm are called micropores [62]. The fact that the average pore diameter of the tested material is 1.903 nm suggests that the porous structure of this activated carbon is dominated by micropores. Micropores are particularly effective in adsorbing small gas and vapor molecules due to strong adsorption interactions in their tight spaces.

For example, in Zhang et al.’s work [61], the results indicate that coconut shell activated carbon has a higher surface area (1556 m^2^ g^−1^ compared to the presented research); however, the pore volume (0.490 cm^3^ g^−1^) and average pore diameter (1.87 nm) are similar. The values of *S_BET_* surface area are also in agreement with the findings of other research studies [57], which found a BET surface area of 824 m^2^ g^−1^ and a total pore volume of 0.502 cm^3^ g^−1^ after carbonization of coconut-shell-based AC above 1000 °C. In turn, Tan et al. [56] produced chemically treated coconut shell-derived AC with a BET specific surface area of 525 m^2^ g^−1^, a total pore volume of 0.291 cm^3^ g^−1^, and an average pore diameter of 14.27, which are significantly different than the values in this study.

**Figure 3:**
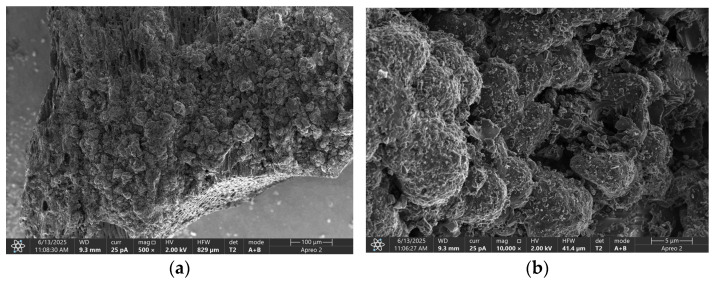

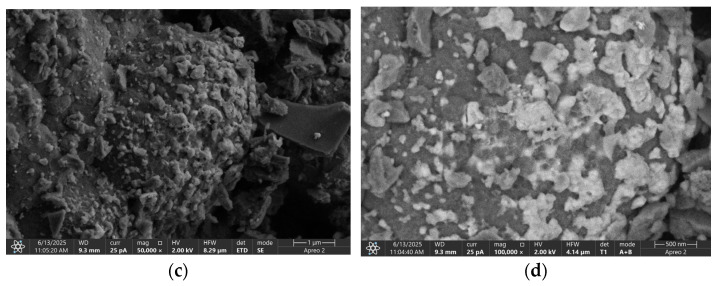
SEM of GAC: (**a**) 500 × magnification, (**b**) 10,000 × magnification, (**c**) 50,000 × magnification, and (**d**) 100,000 × magnification.

Figure 3 presents SEM images of the activated carbon used in this study, characterized by irregular voids. Poorly ordered, folded layers are also visible, indicating the absence of a long-range crystalline structure. At high magnifications, bent, irregular fibrous plates of the carbon layers are visible. The XRD studies confirmed that the tested activated carbon is an amorphous material. The SEM images do not show regular hexagonal graphite planes, which were reflected in a broad background instead of sharp peaks recorded on the diffractogram (Figure 2).

**Figure 4:**
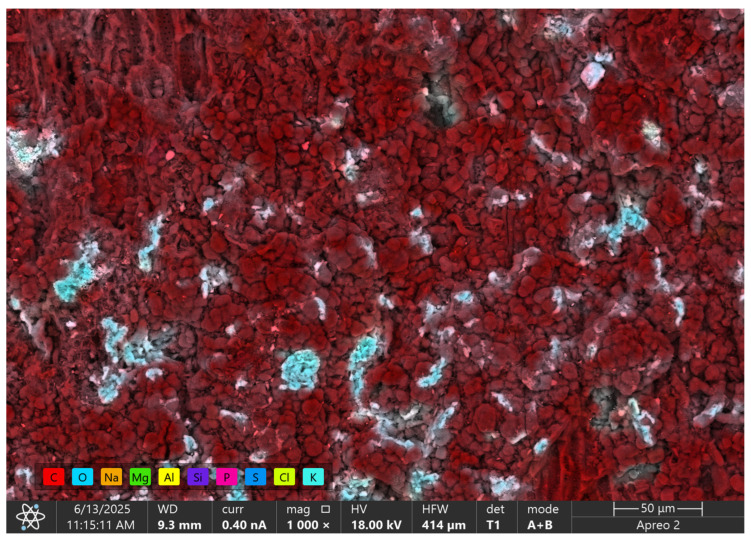
SEM micrograph (magnification 1000×) of the GAC surface with EDS mapping for the main elements.

Figure 4 shows the GAC surface along with the elemental distribution analysis obtained by the EDS method. The dominance of the red color confirms that the carbon material consists mainly of carbon (C), which is consistent with the nature of the sorbent. Significant amounts of oxygen (O), marked in blue, are also visible, indicating the presence of oxygen groups on the surface or in the pores of the carbon, which may affect the adsorption properties, especially in the context of interactions with phosphate and nitrate ions.

Bright clusters of turquoise and light green colors correspond to the presence of chlorine (Cl) and potassium (K), as well as elements such as Na, Si, and Mg. These elements may originate from the material modification process, mineral ash remaining after pyrolysis, or environmental contaminants. Their presence suggests that the surface of GAC has a heterogeneous character, which may facilitate ion sorption processes through additional active centers associated with these elements. It is worth emphasizing that local accumulations of potassium and sodium (visible as bright, concentrated areas) may serve as ion exchange sites, supporting the removal of biogenic components, including phosphorus in the form of orthophosphates.

The tested activated carbon is characterized by a high carbon content and moderate surface oxidation (O/C ~0.16), with a noticeable potassium content (~7.3 wt%), indicating the presence of activation residues or mineral admixtures (Table 4). The results in Table 4 are qualitative in nature because they originate from the EDS analysis. This composition implies a more polar character of the surface and the presence of basic sites associated with K, which may favorably affect the adsorption of polar particles and electrochemical properties, but, at the same time, it suggests the need for possible further purification if very low levels of ash or metals are required.

### 3.2. Column Experiments Results

#### 3.2.1. Effect of GAC Addition on Changes in Nutrient Concentrations

**Figure 5:**
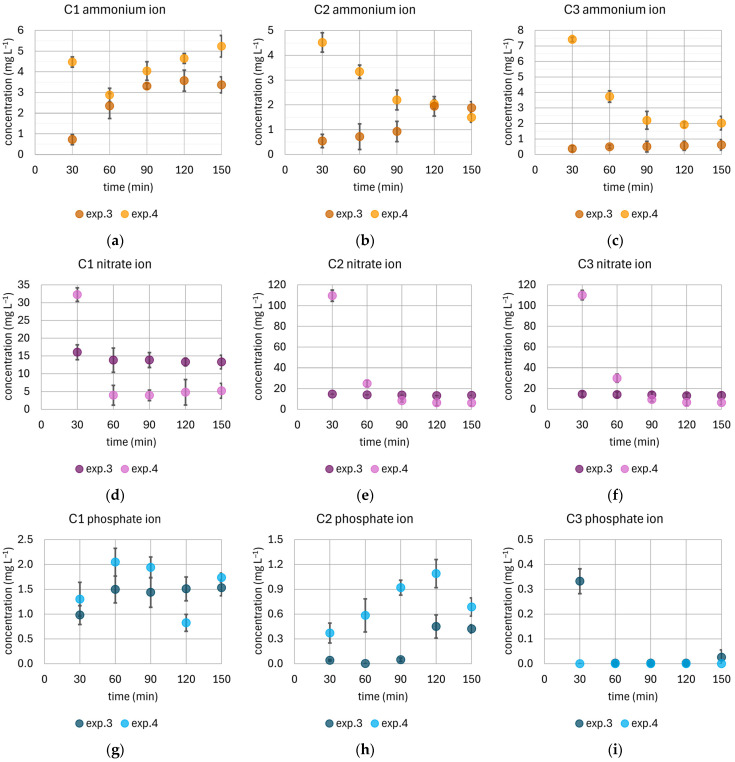
Comparison of changes in the concentrations of selected nutrients during the experiments for less intense rainfall of 120 min: exp.3 without carbon addition and exp.4 with the enrichment in the filter media with a GAC layer. (**a**), (**b**), (**c**) Change in ammonium ion concentration over time for columns C1, C2, and C3, respectively; (**d**), (**e**), (**f**) Change in nitrate ion concentration over time for columns C1, C2, and C3, respectively; (**g**), (**h**), (**i**) Change in phosphate ion concentration over time for columns C1, C2, and C3, respectively. In the calculations for the mean values of phosphate ions for concentrations below the Method Detection Limit, they were replaced by half of this value [63].

By comparing the concentrations determined in the samples during exp.3 and exp.4 (Figure 5), it is possible to assess whether the addition of GAC improves the efficiency of the rain gardens.

The initial concentration of ammonium ions in the model rain was 5 mg L^−1^. In column C1, the addition of GAC did not favorably affect the change in NH_4_^+^ concentration; the values remained relatively stable during the experiment, oscillating around the initial level. In column C2, during exp.3 (without GAC), the use of diatomite resulted in a rapid decrease in NH_4_^+^ concentration to a level of approximately 2 mg L^−1^. Enrichment of the column with GAC caused a slow, systematic decrease in concentration, which led to final values lower than in the variant without modification (exp.3). In the case of column C3, in exp.3, the lowest final NH_4_^+^ concentration among all tested configurations was recorded—it was approximately 0.5 mg L^−1^. The addition of GAC to this column caused an initial increase in NH_4_^+^ concentration to over 7 mg L^−1^, and then, as exp.4 continued, the concentrations decreased to about 2 mg L^−1^.

The initial concentration of nitrate ions in the model rain was 15 mg L^−1^. The introduction of GAC into the columns (exp.4) allowed for obtaining final concentrations lower than in the variant without this modification (exp.3) for each of the columns. However, the GAC addition initially caused an increase in NO_3_^−^ concentration during the first phase of the experiment. The concentrations were 32 mg L^−1^ in column C1 and about 110 mg L^−1^ in columns C2 and C3. In the leachate, after a relatively short period (60 min), a decrease in concentrations was observed, and their values approached the initial model rain. Ultimately, the final NO_3_^−^ concentrations in exp.4 were significantly lower than in exp.3: by 61%, 54%, and 52%, respectively, for C1, C2, and C3.

The assumed concentration for phosphate ions in the model rain was 2.5 mg L^−1^. The addition of GAC in columns C1 and C2 contributed to increasing PO_4_^3−^ concentrations. In column C1, during the experiment without GAC addition, from the 60th minute, the concentration remained stable at around 1.5 mg L^−1^. The final concentration of phosphate ions during the experiment with the filling modification (exp.4) was over 1.5 mg L^−1^ and was 13% higher than the concentration in the experiment without this modification (exp.3). For column C2, this difference was over 60%, but the concentration fluctuated around 0.7 and 0.4 mg L^−1^, respectively, for exp.4 and exp.3. For column C3, although low final PO_4_^3−^, concentrations were obtained in the experiment without GAC addition, the introduction of GAC modification allowed for an even faster reduction in the concentration to <0.005 mg L^−1^ already in the initial phase of the experiment.

#### 3.2.2. Nutrient Removal Efficiency—Influence of Sorbents, Drought, and Variable Rainfall Intensity

##### NH_4_^+^ Removal

The performance of the columns after a drought period can be assessed by comparing the results of experiment 2 (exp.2) with the results of experiments 1 and 3 (exp.1 and exp.3). A long-term (35-day) period without stormwater supply allows for the assessment of whether periods without rainfall affect the effectiveness of nutrient removal (Table 5 and Table 6, comparison of exp.1, exp.2, and exp.3):

- In column C1 with only sand, NH_4_^+^ was removed after the drought period (exp.2) at a similar level as in exp.3 and almost twice as high as in exp.1, which may suggest that rainfall intensity has a greater impact on NH_4_^+^ removal than drying of the filter mixture.
- In the columns with diatomite (C2 and C3), the differences in efficiency were not as significant, but the trends were reversed. After the drought period (exp.2), NH_4_^+^ removal was lower than in exp.1, and in subsequent simulations (exp.3), when the filter media were undried, NH_4_^+^ removal decreased.

The influence of rainfall intensity changes on NH_4_^+^ removal (Table 5—comparison of exp.3 and exp.1) is as follows:

- As mentioned above, in the column without diatomite, less intensive rainfall (exp.3: 105 mL min^−1^) was better purified (reduction 46%) than in experiments with high-intensity rain (exp.1: 310 mL min^−1^) (EMC reduction of 27%).
- In the columns with diatomite, such a tendency was not observed in C2; the average reduction for less intensive rainfall (exp.3) was 77%, while during 30 min rainfall (exp.1), it was 93%. In column C3, the decrease in reduction was not as significant, decreasing from 94 to 90%.

In all columns, the addition of GAC (exp.4) caused the NH_4_^+^ removal to decrease compared to the efficiency of these columns under the same rainfall intensity conditions (exp.3). The NH_4_^+^ reduction was 18% in C1, 44% in C2, and 33% in C3.

##### NO_3_^−^ Removal

The assessment of column performance after a drought period in terms of NO_3_^−^ removal revealed the following:

- In column 1, with only sand after the drought period, the NO_3_^−^ concentration was higher than the inlet concentration, and comparing exp.2 with exp.1 shows that the effectiveness of NO_3_^−^ removal decreased after the drought period.
- In the columns with diatomite (C2 and C3), comparing exp.2 to exp.1 showed an increase in NO_3_^−^ removal efficiency, which may suggest that drying of filter media containing diatomite does not have an adverse effect on NO_3_^−^ removal.

The influence of rainfall intensity change on NO_3_^−^ removal (Table 6, comparison of exp.1 and exp.3) is as follows:

- In the experiments with high-intensity rain (exp.1: 310 mL min^−1^), no removal was observed; on the contrary, NO_3_^−^ concentrations in the effluents were higher than those in synthetic rain. In the column without diatomite, the NO_3_^−^ concentration increased by approximately 11% on average. The columns with diatomite, i.e., C2 and C3, also did not remove NO_3_^−^, and the increase in concentration was even greater: approximately 22% and 15%, on average, for C2 and C3, respectively.
- At lower rain intensity (exp.3: 105 mL min^−1^), NO_3_^−^ reductions were observed, and the effluents from all columns had similarly reduced concentrations—6–8% in EMC. The influence of GAC addition on NO_3_^−^ removal (Table 6, comparison of exp.3 and exp.4) is as follows:
- In the column without diatomite (C1), the addition of carbon (under low rainfall intensity conditions) significantly improved NO_3_^−^ removal to an average level of about 39%.
- In the columns with diatomite (C2 and C3), no improvement in NO_3_^−^ removal was observed due to the addition of GAC. NO_3_^−^ concentrations significantly increased compared to inlet concentrations to 90 and 97%.

##### N Removal (The Sum of N-NO_3_^−^ and N-NH_4_^+^)

**Figure 6:**
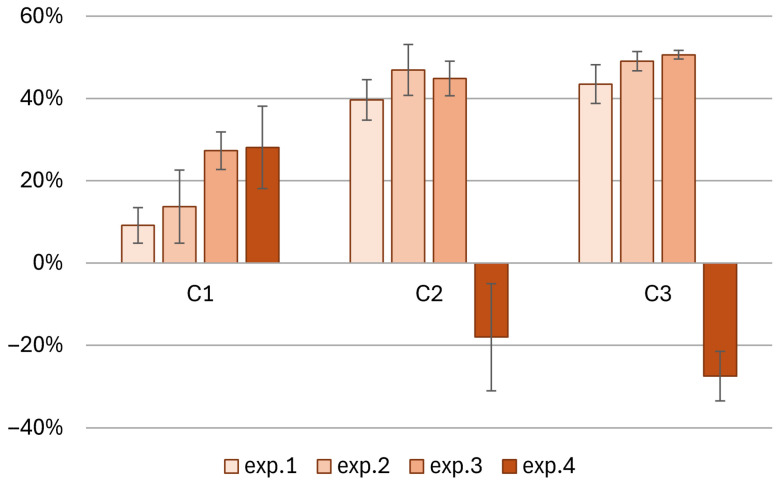
Effectiveness of nitrogen load removal (N-NO_3_^−^ and N-NH_4_^+^) in individual columns during the experiments.

The effectiveness of the columns during the individual experiments was also assessed through the analysis of nitrogen load removal (as the sum of N-NO_3_^−^ and N-NH_4_^+^), which is shown in Figure 6. In column 1, the lowest nitrogen removal efficiency was obtained in the first experiment (exp.1) and the highest in the fourth (exp.4). In the remaining experiments, the efficiency of the columns increased, meaning that changes in factors such as lower rainfall intensity (exp.2, exp.3) or substrate desiccation (exp.2) may positively affect N removal. The addition of GAC (exp.4) in C1 slightly increased the nitrogen removal efficiency. In columns C2 and C3, the highest nitrogen removal efficiency, 45–51%, was obtained in experiments 2 and 3 (exp.2 and exp.3). In experiment 4 (exp.4), with the addition of GAC, a significant increase in the nitrogen concentration was observed in the water effluent from columns C2 and C3. The column efficiency was negative, indicating nitrogen leaching from the columns.

##### PO_4_^3−^ Removal

**Figure 7:**
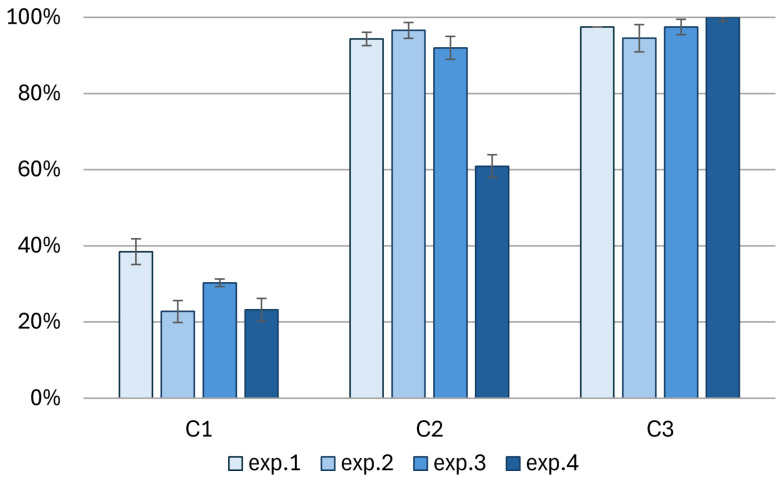
Effectiveness of phosphorus load removal (P-PO_4_^3−^) in individual columns during the experiments.

Phosphorus removal was always lowest in column C1, regardless of the experimental conditions (Figure 7). The addition of diatomite in column C2 increased the phosphorus removal efficiency, reaching an efficiency above 90%. It is worth noting that in columns C1 and C2, phosphorus removal in experiment 4 (exp.4—GAC addition) decreased compared to experiment 3 (exp.3) and was 23% and 61%, respectively. In column C3, the lowest efficiency of phosphorus removal was 95% during experiment 2 (exp.2). The addition of GAC in this column improved the efficiency of phosphorus removal, enabling complete removal of this nutrient.

The phosphorus removal performance of the garden after the drought period in experiment 2 (exp.2) was similar to that in experiments 1 and 3 (exp.2 and exp.3). However, it is worth noting that in the case of column C1 (without diatomite), phosphorus removal in the dried column was the lowest (Figure 7).

Lower rainfall intensity did not improve PO_4_^3−^ reduction (Figure 7—comparison of exp.1 and exp.3). In C1, phosphorus removal decreased from 38 to 30%, while in the other columns, it remained practically unchanged.

The influence of GAC addition on PO_4_^3−^ removal (Figure 7—comparison of exp.1 and exp.3) is as follows:

- In column C1, GAC addition in exp.4 resulted in a PO_4_^3−^ reduction at around 23%, which was significantly lower than in exp.3 with the same precipitation parameters, where a reduction of 30% was achieved.
- In column C2, PO_4_^3−^ reduction was significantly lower at 61%, while in the other experiments, it was over 90%.
- In column C3, PO_4_^3−^ reduction was complete, but in the other experiments, it was also very high, at 97–98%.

## 4. Discussion

The N and P removal efficiencies achieved with GAC addition (comparison of efficiencies in exp.3 and exp.4 in column C1) show that carbon addition improves rain garden performance. The removal of P increased from 30 to 73% after adding GAC; in the range of N removal (the sum of N-NO_3_^−^ and N-NH_4_^+^), the reduction was at a similar level both in the column without and with the addition of GAC (27% and 28%, respectively). However, the removal of the individual tested ions changed: GAC addition worsened NH_4_^+^ removal but improved NO_3_^−^ removal.

The obtained results in terms of N removal compared to other studies (Table 7) are more favorable, e.g., Kus et al. [38] showed that the addition of GAC (in sand and GAC proportions of 10:1) ensured TN removal at the level of 4–14%. Some studies, e.g., Kang et al. [42], in columns with the addition of activated carbon (AC produced from selected coal by a high-temperature steam activation process) showed a higher ability to adsorb nitrate up to 41%. Yuea et al. [39] also showed higher NO_3_^−^ reduction by 38–72% depending on the thickness of the AC layer (sewage sludge-based activated carbon). Huang et al. [57] studied NH_4_^+^ removal by adding activated carbon (produced from discarded coconut shells) in columns and showed a reduction in the leachate by about 10% (a similar level of 18% NH_4_^+^ removal was obtained in exp.4 in column C1 after adding GAC). Also, studies conducted by Liu et al. [25] confirmed the effectiveness of using AC as a supporting layer for stormwater treatment, achieving TN removal efficiency of 72–85%. In laboratory studies of a rain garden, Zhang et al. [27] showed that columns filled with 94% garden soil and 6% AC or biochar are able to partially remove nitrogen from stormwater. The removal of total nitrogen (TN) for both types of additives reached an average efficiency of 50% [27]. The obtained values are difficult to compare with our experiments because the columns described by Zhang et al. [27], unlike ours, had vegetation. In general, vegetation supports the removal of nutrients, but, as reported by Bratieres et al. [64], there are significant differences between species.

In terms of P removal, a similar effectiveness of using AC as a supporting layer for stormwater treatment was demonstrated by Liu et al. [25], achieving a TP reduction of 60–92%, as well as by Zhang et al. [27], achieving a reduction of approximately 60–80%. A lower phosphorus reduction level was shown by Kus et al. [38], who demonstrated that the addition of GAC (in sand and GAC proportions of 10:1) provided phosphorus removal at a level of 11%; Huang et al., who used AC (produced from discarded coconut shells) in columns and achieved a reduction of about 20%; and Ma et al. [31], who showed a reduction in orthophosphate concentrations at the level of 33%. According to Ma et al. [31], GAC removed phosphorus very poorly in the range of 20–30% at volumes of 10–20 L.

In the conducted experiments, it was shown that long-term drying (3.5 months) may affect the ability to remove N (the sum of N-NO_3_^−^ and N-NH_4_^+^), but unfavorable trends (leaching of N from the filter mixture) are significant for the column without diatomite (C1, exp.2 removal: −26%; exp.3 removal: −6%). In the columns with diatomite, i.e., C2 and C3, after the drought period, removal of 23–26% was achieved, followed by a very similar level of 20–28%. In the case of phosphorus, removal after the dry period and in the wet period did not undergo such significant changes (dry period: 23, 97, and 95%; wet period: 30, 92, and 97%). The effectiveness of operation during wet and dry periods was presented, among others, in review articles [21,66]. Biswal et al. [21] cited the results of four studies on nitrogen removal in wet/dry conditions and drew the general conclusion that wet conditions mainly support denitrification and dry conditions support nitrification and ammonification. Laurenson et al. [66] cited, in turn, three previous studies [67,68,69], in which significant increases in NO_3_^−^ concentration were observed after longer periods of drought. Søberg et al. [70] studied the impact of dry and wet periods (and other factors affecting N removal capacity) and showed trends similar to those mentioned above, i.e., an increase in NO_x_-N and TN concentrations with increasing length of the preceding dry period. However, the values of the reductions obtained are not comparable to our studies due to the use of plant plantings in columns and the saturation zone. Similar studies were also conducted by Yu et al. [71], who carried out experiments in columns with plantings and a variable height of the submerged zone during rainfall periods and variable length dry periods (from 0.5 to 4 days). Under the experimental conditions used, it was shown that the N removal efficiency was the lowest after longer periods without rainfall (4 days).

The conducted research also concerned the assessment of the influence of rainfall intensity on the efficiency of nutrient removal. Comparison of the results of exp.1 and exp.3 allows for the conclusion that with lower rainfall intensity, the columns remove N significantly better, while in the case of P, no such tendency is visible. Similar results were obtained by Zhang et al. [27], who tested rainfall of the same duration (2 h) but with different return periods (from 2 to 50 yr) and, therefore, different amounts/intensities. They showed that total nitrogen (TN) removal for filtration media with GAC achieved an average efficiency of 50% regardless of the return periods, while total phosphorus (TP) removal efficiency decreased with increasing return periods: from about 80% for rainfall of 2 yr and 54.71 mm to about 60% for rainfall of 50 yr and 115 mm. The study indicates that the pollutant removal efficiency is variable and strongly depends on the precipitation characteristics and the initial conditions in the filter material, including saturation. The influence of rainfall intensity on the efficiency of N removal was also studied by Lopez-Ponnada et al. [72], who reached similar conclusions: at a lower intensity (4.1 cm h^−1^), TN removal was approximately 60%, while at an intensity of 13.9 cm h^−1^, it was approximately 35%.

## 5. Conclusions

Rain gardens are an effective tool for managing stormwater in cities, reducing both surface runoff and pollutant load. The variability in precipitation conditions (intense rainfall, periods of drought) significantly affects the efficiency of the system, which should be taken into account when designing a rain garden. Optimizing the composition of the bed by using sorbent materials increases the durability and environmental safety of rain gardens.

1. The GAC additive did not improve phosphate removal in a column without diatomite, as evidenced by the results of experiment 4 for column C1. In the same experiment, also for column C1, it was observed that the presence of GAC caused a 2.4-fold deterioration in ammonium ion removal while increasing the nitrate ion removal efficiency by 6.5-fold.
2. The combination of diatomite and GAC did not contribute to the removal of total nitrogen and phosphates. Nitrogen, considered as the sum of NO_3_^−^ and NH_4_^+^ ions, was removed at the same level regardless of the addition of GAC. Phosphate removal using diatomite alone was very high (exp.3 for C2 and C3), while the addition of GAC caused a 1.5-fold deterioration in column performance, which was observed in C2 during exp.4. In the case of column C3 in exp.3, without GAC addition, almost complete phosphorus removal occurred up to 60 min, while the addition of GAC shortened this time to 30 min. Nevertheless, it was primarily diatomite that was responsible for the effective removal of phosphates, achieving an efficiency close to 100%.
3. Our experiments confirmed the results of other researchers, suggesting that drought periods can impair nitrogen removal, as demonstrated in C1. Analysis of the impact of various rainfall scenarios showed that short-term, intense rainfall reduced pollutant removal efficiency, primarily due to reduced water contact time with the filter substrate. This effect was most noticeable for nitrogen, while phosphorus removal remained relatively stable due to sorption processes. Variants with sorption materials showed less sensitivity to variable hydraulic conditions.
4. The change in rainfall intensity, tested in 30 min and 2 h variants, did not have a significant impact on phosphorus removal efficiency. Columns C2 and C3 were characterized by similarly high efficiency, while slightly worse results were observed in column C1. For nitrogen, an increase in removal efficiency was observed with less intense rainfall, which can be associated with a longer contact time of rainwater with the filtration material in the columns.
5. Referring to the criteria described in the table in the introduction (Table 1), the columns studied by the authors meet criterion 2, and the others are as follows:
  - Water retention—the focus was on water quality, not flow quantity.
  - Pollutant removal:
    − Very good (>60%) for phosphorus (C2 and C3, exp.1–4);
    − Moderate (from 30 to 60%) for phosphorus (C1, exp.1 and exp.3) and nitrogen (C2 and C3, exp.1–3);
    − Poor (< 30%) for phosphorus (C1; exp.2 and exp.4) and nitrogen (C1, exp.1–4; C2, exp.4; and C3, exp.4).
  - Safety (leaching) was not measured directly. In the future, the authors will focus on the mechanisms of nutrient removal. Based on their experiments, the authors conclude that using only GAC is safe and does not cause nutrient leaching. Combining both sorbents (GAC and diatomite) increases nitrate concentrations (exp.4, C2 and C3).

The research results can serve as a basis for developing design and operational guidelines for rain gardens with the addition of sorbents (e.g., diatomite and GAC) in urban conditions, taking into account periodic droughts and varied rainfall intensities.

## Figures and Tables

**Table 1:** Evaluation of materials used in the construction of rain gardens according to the three criteria adopted for analysis.

No.	Material	Criteria			References
		1	2	3	
1	gravel (20 cm), river sand (33 cm), soil (33 cm)	yes	yes	yes	[8]
2	mixture of sand to soil medium consists of 90.6% sand, 6.9% silt, and 2.5% clay with 0.7% organic matter	no	yes	no	[28]
3	gravel + mixture of river sand and dolomite (10:1, *w*/*w*)	N/A	yes	N/A	[41]
4	dolomite—20 cm, sand and diatomite (2:1)—45 cm, gravel—5 cm	no	yes	no	[7]
5	soil (40 cm), sand (40 cm), and bottom ash (40 cm)	yes	yes	yes	[6]
6	sandy loam soil, mulch	no	yes	no	[9]
7	mulch (bark) 50 mm, soil 250 mm (clay content of red soil was above 40%), sand filter 100 mm, gravel drainage 200 mm (the particle size of the gravel was 20–30 mm)	yes	yes	yes	[26]
8	8–27 cm layer of 11 types of fillers, including 5 natural materials, 3 industrial wastes, and 3 artificial materials	yes	yes	yes	[32]
9	gravel (30 cm), soil with 25% expanded shale aggregate (50 cm), hardwood mulch (10 cm)	yes	yes	no	[34]
10	3 cm gravel, 20 cm sand, and 5 cm tea leaf waste or wood chips or coconut husk	yes	yes	N/A	[37]
11	pea gravel + sandy media (90% sand and 10% Sphagnum peat moss) + wood chips (20 cm) + sandy media	yes	yes	yes	[33]
12	gravel, fine sand, sorption material: sand, biochar, Sphagnum peat or ash, sandy loam mixed with pumice stone and a sorption material, sandy loam mixed with pumice stone	yes	yes	no	[13]
13	biochar 22.9 cm + pea gravel 2 × 7.6 cm	N/A	yes	N/A	[35]
14	mix: gravel + sand + soil + 4% rice husk biochar or iron-coated biochar	N/A	yes	N/A	[36]
15	gravel (10 cm), sand with 5% woodchips and 5% sugarcane bagasse (*V*/*V*) (20 cm), soil with 10% biochar (*V*/*V*) (40 cm), 5–10 cm crumb of pine bark	yes	yes	yes	[17]
16	mix: sand + pumice + activated char or biochar	yes	yes	N/A	[42]
17	gravel (10 cm), media layer (70 cm): 94% garden soil + 6% activated carbon or biochar in the upper or biochar in the lower	yes	yes	yes	[27]
18	gravel (5 cm), media layer (2.5 cm): silica sand, gravel, zeolite, activated carbon, or slag, sail layer (22.5 cm), bark (12.5 cm)	N/A	yes	yes	[25]
19	GAC, zeolite, an engineered media	N/A	yes	N/A	[31]
20	mix: soil + 0.3% GAC	N/A	yes	N/A	[10]
21	mix: GAC + sand (1:1 and 1:10)	N/A	yes	N/A	[38]
22	SBAC—sludge-based activated carbon	no	yes	no	[39]

Criteria: 1. Water retention capacity, 2. pollutant removal capability, and 3. safety for the aquatic environment; yes—confirmation that the given criterion is met, no—failure to meet the given criterion, and N/A—not available.

**Table 2:** Characteristics of ion-selective electrodes [52].

	Nitrate Electrode	Ammonium Electrode
Detection range	0.6–6000 mg L^−1^	0.9–9000 mg L^−1^
Operating temperature range	0–40 °C	0–40 °C
Required solution pH	2–12	1–8
Half-cell	Ag/AgCl	Ag/AgCl
Reference electrode	Chloride-selective with a double junction filled with 1 M L^−1^ KCl and 1 M L^−1^ (NH_4_)_2_SO_4_	Chloride-selective with a double junction filled with 1 M L^−1^ KCl and 0.1 M L^−1^ CH_3_COOLi
Required ISA ionic strength buffer	Yes (2 mL/100 mL sample)	Yes (25 mL/25 mL sample)
Interfering ions	K^+^, Rb^+^, H^+^, Cs^+^, Li^+^, Na^+^	ClO_4_^−^

**Table 3:** Porous texture parameters of GAC.

Sample	BET Surface (*S_BET_*, m^2^ g^−1^)	Total Pore Volume (*V_total_*, cm^3^ g^−1^)	Average Pore Diameter (*D_A_*, nm)
Activated carbon	872.77	0.415	1.903

**Table 4:** Elemental composition of GAC.

Element	C	O	Na	Mg	Al	Si	P	S	Cl	K
Atomic %	83.1	13.5	0.1	0.1	0.1	0.1	0.1	0.2	0.1	2.5

**Table 5:** NH_4_^+^ removal.

Experiment	C1	C2	C3
exp.1	27%	93%	94%
exp.2	43%	84%	92%
exp.3	46%	77%	90%
exp.4	18%	44%	33%

**Table 6:** NO_3_^−^ removal (minus means that there has been an increase).

Experiment	C1	C2	C3
exp.1	−11%	−22%	−15%
exp.2	−20%	4%	0%
exp.3	6%	8%	7%
exp.4	39%	−90%	−97%

**Table 7:** Examples of the effectiveness of N and P removal after GAC application.

Amount of GAC Additive	N Removal (%)	P Removal (%)	Reference
1 g L^−1^ leachate	72% NO_3_-N (initial concentration 2 mg L^−1^), synthetic rain from distilled water enriched with nutrients	41% PO_4_-P (initial concentration 1 mg L^−1^), synthetic rain from distilled water enriched with nutrients	[39]
1 g L^−1^ leachate	38% for NO_3_-N leachate from stormwater	20% for PO_4_-P leachate from stormwater	
3 g L^−1^ leachate	72% for NO_3_-N leachate from stormwater	31% for PO_4_-P leachate from stormwater	
18 cm horizontal column completely filled with GAC	not examined	33%	[31]
20 g L^−1^ in synthetic model solution	17% removal from synthetic model solution	86% removal from synthetic model solution	[65]
two experiments: GAC and sand (ratio of 1:1), GAC and sand (ratio of 1:10)	7–14% TN	11% TP	[38]
2.5 cm GAC layer	72–85% TN	60–92% TP	[25]
10.2 cm GAC layer	not examined	20–30%	[35]
70 cm filtration layer with 6% GAC	22–52% TN 55–72% NH_4_^+^-N 16–44% NO_3_^−^-N	60–82% TP 60–82% PO_4_^3−^-P	[27]

## Data Availability

The original contributions presented in this study are included in the article. Further inquiries can be directed to the corresponding author.

## Abbreviations

The following abbreviations are used in this manuscript:

AC	activated carbon
ASAP	Accelerated Surface Area and Porosimetry system
BET	Brunauer–Emmett–Teller method
BJH	Barrett–Joyner–Halenda method
C1, C2, C3	designations of columns
COD	chemical oxygen demand
EDS	Energy Dispersive X-ray Spectroscopy
EMC	event mean concentration
exp.1	experiment 1 described in this article
exp.2	experiment 2 described in this article
exp.3	experiment 3 described in this article
exp.4	experiment 4 described in this article
GAC	granular activated carbon
ICDD	International Centre for Diffraction Data
ISA	Ionic Strength Adjuster
IUPAC	International Union of Pure and Applied Chemistry
LFF	Long Fine Focus
NBSs	Nature-Based Solutions
PDF	Powder Diffraction File
SBAC	sludge-based activated carbon
*S_BET_*	surface areas calculated using the Brunauer–Emmett–Teller BET method
SEM	scanning electron microscope
TN	total nitrogen
TP	total phosphorus
XRD	X-ray diffraction

## References

[1] European Commission Nature-Based Solutions. https://research-and-innovation.ec.europa.eu/research-area/environment/nature-based-solutions_en.

[2] Kuller M., Bach P.M., Ramirez-Lovering D., Deletic A. (2017). Framing Water Sensitive Urban Design as Part of the Urban Form: A Critical Review of Tools for Best Planning Practice. Environ. Model. Softw..

[3] Bak J., Barjenbruch M. (2022). Benefits, Inconveniences, and Facilities of the Application of Rain Gardens in Urban Spaces from the Perspective of Climate Change—A Review. Water.

[4] Osheen, Singh K.K., Agnihotri A.K., Bansal A., Reddy K. (2019). Rain Garden—A Solution to Urban: A Review. Lecture Notes in Civil Engineering Sustainable Engineering Proceedings of EGRWSE 2018.

[5] Nowogoński I. (2021). Runoff Volume Reduction Using Green Infrastructure. Land.

[6] Jeon M., Guerra H.B., Choi H., Kwon D., Kim H., Kim L.H. (2021). Stormwater Runoff Treatment Using Rain Garden: Performance Monitoring and Development of Deep Learning-Based Water Quality Prediction Models. Water.

[7] Grela A., Łach M., Pamuła J., Łach K., Godyń I., Malina D., Wzorek Z., Setlak K., Grela D. (2024). Effect of Diatomite Application on the Removal of Biogenic Pollutants in Rain Gardens. Materials.

[8] Davis A.P., Shokouhian M., Sharma H., Minami C. (2006). Water Quality Improvement through Bioretention Media: Nitrogen and Phosphorus Removal. Water Environ. Res..

[9] Davis A.P., Shokouhian M., Sharma H., Minami C. (2001). Laboratory Study of Biological Retention for Urban Stormwater Management. Water Environ. Res..

[10] Ekanayake D., Loganathan P., Johir M.A.H., Kandasamy J., Vigneswaran S. (2021). Enhanced Removal of Nutrients, Heavy Metals, and PAH from Synthetic Stormwater by Incorporating Different Adsorbents into a Filter Media. Water Air Soil. Pollut..

[11] Hong E., Seagren E.A., Davis A.P. (2006). Sustainable Oil and Grease Removal from Synthetic Stormwater Runoff Using Bench-Scale Bioretention Studies. Water Environ. Res..

[12] Nocoń W., Moraczewska-Majkut K., Wiśniowska E. (2018). Microplastics in Surface Water under Strong Anthropopression. Desalination Water Treat..

[13] Johansson G., Fedje K.K., Modin O., Haeger-Eugensson M., Uhl W., Andersson-Sköld Y., Strömvall A.M. (2024). Removal and Release of Microplastics and Other Environmental Pollutants during the Start-up of Bioretention Filters Treating Stormwater. J. Hazard. Mater..

[14] Barbosa A.E., Fernandes J.N., David L.M. (2012). Key Issues for Sustainable Urban Stormwater Management. Water Res..

[15] Withers P.J.A., Neal C., Jarvie H.P., Doody D.G. (2014). Agriculture and Eutrophication: Where Do We Go from Here?. Sustainability.

[16] Glibert P.M. (2017). Eutrophication, Harmful Algae and Biodiversity—Challenging Paradigms in a World of Complex Nutrient Changes. Mar. Pollut. Bull..

[17] Wang H., Sun Y., Zhang L., Wang W., Guan Y. (2021). Enhanced Nitrogen Removal and Mitigation of Nitrous Oxide Emission Potential in a Lab-Scale Rain Garden with Internal Water Storage. J. Water Process Eng..

[18] Smol M., Włóka D. (2022). Use of Natural Sorbents in the Processes of Removing Biogenic Compounds from the Aquatic Environment. Sustainability.

[19] Tian J., Jin J., Chiu P.C., Cha D.K., Guo M., Imhoff P.T. (2019). A Pilot-Scale, Bi-Layer Bioretention System with Biochar and Zero-Valent Iron for Enhanced Nitrate Removal from Stormwater. Water Res..

[20] Sharma R., Malaviya P. (2021). Management of Stormwater Pollution Using Green Infrastructure: The Role of Rain Gardens. Wiley Interdiscip. Rev. Water.

[21] Biswal B.K., Vijayaraghavan K., Adam M.G., Lee Tsen-Tieng D., Davis A.P., Balasubramanian R. (2022). Biological Nitrogen Removal from Stormwater in Bioretention Cells: A Critical Review. Crit. Rev. Biotechnol..

[22] Zhang L., Deng F., Liu Z., Ai L. (2021). Removal of Ammonia Nitrogen and Phosphorus by Biochar Prepared from Sludge Residue after Rusty Scrap Iron and Reduced Iron Powder Enhanced Fermentation. J. Environ. Manag..

[23] Yin H., Zhang M., Huo L., Yang P. (2022). Efficient Removal of Phosphorus from Constructed Wetlands Using Solidified Lanthanum/Aluminum Amended Attapulgite/Biochar Composite as a Novel Phosphorus Filter. Sci. Total Environ..

[24] Pugliese L., Canga E., Hansen H.C.B., Kjærgaard C., Heckrath G.J., Poulsen T.G. (2023). Long-Term Phosphorus Removal by Ca and Fe-Rich Drainage Filter Materials under Variable Flow and Inlet Concentrations. Water Res..

[25] Liu A., Jiang Y., Dockko S., Guan Y. (2015). Characterizing Stormwater Treatment Efficiency at the Laboratory Scale for Effective Rain Garden Design. Desalination Water Treat..

[26] Chen C., Li Y., Le W., You C., Liu Z., Liu W., Zhang R. (2023). Field Performance of Rain Garden in Red Soil Area in Southern China. Water.

[27] Zhang W., Sang M., Sun H., Che W., Li J. (2021). Influence of Rainfall on the Performance of Bioretention Systems Modified with Activated Carbon and Biochar. J. Hydro-Environ. Res..

[28] Yang H., McCoy E.L., Grewal P.S., Dick W.A. (2010). Dissolved Nutrients and Atrazine Removal by Column-Scale Monophasic and Biphasic Rain Garden Model Systems. Chemosphere.

[29] Dietz M.E., Clausen J.C. (2005). A Field Evaluation of Rain Garden Flow and Pollutant Treatment. Water Air Soil Pollut..

[30] LeFevre G.H., Paus K.H., Natarajan P., Gulliver J.S., Novak P.J., Hozalski R.M. (2015). Review of Dissolved Pollutants in Urban Storm Water and Their Removal and Fate in Bioretention Cells. J. Environ. Eng..

[31] Ma J., Lenhart J.H., Tracy K. (2011). Orthophosphate Adsorption Equilibrium and Breakthrough on Filtration Media for Storm-Water Runoff Treatment. J. Irrig. Drain. Eng..

[32] Huang T., Wang Z., Nie Y., Liu H., Li P., Yang J., Wu B. (2025). Efficiently Optimized Multi-Fillers for Rain Gardens: Long-Term Pollution Control Performance. Water Cycle.

[33] Gilchrist S., Borst M., Stander E.K. (2014). Factorial Study of Rain Garden Design for Nitrogen Removal. J. Irrig. Drain. Eng..

[34] Strong P., Hudak P.F. (2015). Nitrogen and Phosphorus Removal in a Rain Garden Flooded with Wastewater and Simulated Stormwater. Environ. Qual. Manag..

[35] Reddy K.R., Xie T., Dastgheibi S. (2014). Evaluation of Biochar as a Potential Filter Media for the Removal of Mixed Contaminants from Urban Storm Water Runoff. J. Environ. Eng..

[36] Xiong J., Ren S., He Y., Wang X.C., Bai X., Wang J., Dzakpasu M. (2019). Bioretention Cell Incorporating Fe-Biochar and Saturated Zones for Enhanced Stormwater Runoff Treatment. Chemosphere.

[37] Husna T., Aminuddin A.G., Nor Azazi Z. (2014). The Impact of Stormwater Runoff on Nutrient Removal in Sand Columns. Appl. Mech. Mater..

[38] Kus B., Kandasamy J. (2009). Low-Cost Filtration System to Treat First-Flush Stormwater. Water Air Soil Pollut. Focus..

[39] Yue C., Li L.Y., Johnston C. (2018). Exploratory Study on Modification of Sludge-Based Activated Carbon for Nutrient Removal from Stormwater Runoff. J. Environ. Manag..

[40] Wang M., Zhuang J., Sun C., Wang L., Zhang M., Fan C., Li J. (2024). The Application of Rain Gardens in Urban Environments: A Bibliometric Review. Land.

[41] Prochaska C.A., Zouboulis A.I. (2006). Removal of Phosphates by Pilot Vertical-Flow Constructed Wetlands Using a Mixture of Sand and Dolomite as Substrate. Ecol. Eng..

[42] Kang J., Davila M., Mireles S., Ho J. (2017). Nitrate Leaching from Sand and Pumice Geomedia Amended with Pyrogenic Carbon Materials. Environments.

[43] Marvin J.T., Passeport E., Drake J. (2020). State-of-the-Art Review of Phosphorus Sorption Amendments in Bioretention Media: A Systematic Literature Review. J. Sustain. Water Built Environ..

[44] Czerniakowski Z.W., Gargała-Polar M. (2020). Ogrody Deszczowe Jako Sposób Retardacji Strat Wody Opadowej w Terenach Zieleni Miejskiej. Pol. J. Sustain. Dev..

[45] U.S. Environmental Protection Agency (2013). Stormwater to Street Trees: Engineering Urban Forests for Stormwater Management. https://www.epa.gov/sites/default/files/2015-11/documents/stormwater2streettrees.pdf.

[46] Jiang C., Wang T., Wu X., Dang Z., Li H. (2025). Replacement Depth and Lifespan Prediction of Enhanced Bioretention Media under TSS Impact Conditions. Environ. Technol..

[47] Kasprzyk M., Szpakowski W., Poznańska E., Boogaard F.C., Bobkowska K., Gajewska M. (2022). Technical Solutions and Benefits of Introducing Rain Gardens—Gdańsk Case Study. Sci. Total Environ..

[48] Kravchenko M., Trach Y., Trach R., Tkachenko T., Mileikovskyi V. (2024). Improving the Efficiency and Environmental Friendliness of Urban Stormwater Management by Enhancing the Water Filtration Model in Rain Gardens. Water.

[49] Kabekkodu S.N., Dosen A., Blanton T.N. (2024). PDF-5+: A comprehensive Powder Diffraction FileTM for materials characterization. Powder Diffr..

[50] (2006). Water Quality—Determination of Phosphorus—Ammonium Molybdate Spectrometric Method.

[51] International Standards Water Quality-Determination of Phosphorus-Ammonium Molybdate Spectrometric Method. https://cdn.standards.iteh.ai/samples/36917/7f66bff84db146e398013e961ce7ab4c/ISO-6878-2004.pdf.

[52] Detektor Elektordy Laboratoryjne. http://www.detektor.biz/strony/elektrody_rodzaje.php.

[53] Elmetron Ph/Jonometr CPI-601. https://elmetron.com.pl/CPI-601.html.

[54] Elmetron Laboratoryjny Przyrząd Wielofunkcyjny CX-505. https://elmetron.com.pl/CX-505.html.

[55] Yahya M.A., Al-Qodah Z., Ngah C.W.Z. (2015). Agricultural Bio-Waste Materials as Potential Sustainable Precursors Used for Activated Carbon Production: A Review. Renew. Sustain. Energy Rev..

[56] Tan I.A.W., Abdullah M.O., Lim L.L.P., Yeo T.H.C. (2017). Surface Modification and Characterization of Coconut Shell-Based Activated Carbon Subjected to Acidic and Alkaline Treatments. J. Appl. Sci. Process Eng..

[57] Huang P.H., Cheng H.H., Lin S.H. (2015). Adsorption of Carbon Dioxide onto Activated Carbon Prepared from Coconut Shells. J. Chem..

[58] Yang J., Han S. (2018). Kinetics and Equilibrium Study for the Adsorption of Lysine on Activated Carbon Derived from Coconut Shell. Desalination Water Treat..

[59] Asada T., Oikawa K., Kawata K., Ishihara S., Iyobe T., Yamada A. (2004). Study of Removal Effect of Bisphenol A and .BETA.-Estradiol by Porous Carbon. J. Health Sci..

[60] Hu Z., Srinivasan M.P., Ni Y. (2001). Novel Activation Process for Preparing Highly Microporous and Mesoporous Activated Carbons. Carbon.

[61] Zhang L., Tu L.Y., Liang Y., Chen Q., Li Z.S., Li C.H., Wang Z.H., Li W. (2018). Coconut-Based Activated Carbon Fibers for Efficient Adsorption of Various Organic Dyes. RSC Adv..

[62] (2025). ‘Micropore’ in IUPAC Compendium of Chemical Terminology.

[63] Commission Directive 2009/90/EC of 31 July 2009 Laying Down, Pursuant to Directive 2000/60/EC of the European Parliament and of the Council, Technical Specifications for Chemical Analysis and Monitoring of Water Status. https://eur-lex.europa.eu/eli/dir/2009/90/oj/eng.

[64] Bratieres K., Fletcher T.D., Deletic A., Zinger Y. (2008). Nutrient and Sediment Removal by Stormwater Biofilters: A Large-Scale Design Optimisation Study. Water Res..

[65] Wystalska K., Grosser A. (2024). Sewage Sludge-Derived Biochar and Its Potential for Removal of Ammonium Nitrogen and Phosphorus from Filtrate Generated during Dewatering of Digested Sludge. Energies.

[66] Laurenson G., Laurenson S., Bolan N., Beecham S., Clark I. (2013). The Role of Bioretention Systems in the Treatment of Stormwater. Advances in Agronomy.

[67] Hatt B.E., Fletcher T.D., Deletic A. (2007). Hydraulic and Pollutant Removal Performance of Stormwater Filters under Variable Wetting and Drying Regimes. Water Sci. Technol..

[68] Hatt B.E., Fletcher T.D., Deletic A. (2009). Hydrologic and Pollutant Removal Performance of Stormwater Biofiltration Systems at the Field Scale. J. Hydrol..

[69] Kim H., Seagren E.A., Davis A.P. (2003). Engineered Bioretention for Removal of Nitrate from Stormwater Runoff. Water Environ. Res..

[70] Søberg L.C., Viklander M., Blecken G.T. (2021). Nitrogen Removal in Stormwater Bioretention Facilities: Effects of Drying, Temperature and a Submerged Zone. Ecol. Eng..

[71] Yu S., He K., Xia C., Qin H. (2023). Modeling the Effect of the Submerged Zone on Nitrogen Removal Efficiency of a Bioretention System under Dry-Wet Alterations. J. Hydrol..

[72] Lopez-Ponnada E.V., Lynn T.J., Ergas S.J., Mihelcic J.R. (2020). Long-Term Field Performance of a Conventional and Modified Bioretention System for Removing Dissolved Nitrogen Species in Stormwater Runoff. Water Res..

